# Early Initiation of Extracorporeal Blood Purification Using the AN69ST (oXiris®) Hemofilter as a Treatment Modality for COVID-19 Patients: a Single-Centre Case Series

**DOI:** 10.21470/1678-9741-2020-0403

**Published:** 2022

**Authors:** Petar Ugurov, Dijana Popevski, Tanja Gramosli, Dashurie Neziri, Dragica Vuckova, Marko Gjorgon, Emil Stoicovski, Sanja Marinkovic, Lidija Veljanovska-Kiridjievska, Katerina Ignevska, Sanja Mehandziska, Elena Ambarkova, Zan Mitrev, Rodney Alexander Rosalia

**Affiliations:** 1 Zan Mitrev Clinic, Republic of North Macedonia.

**Keywords:** COVID-19, Interleukin-6, Heparin, TNF protein, human, Tumor Necrosis Factor-alpha, Respiratory Distress Syndrome, Adult, C-Reactive Protein, Blood Platets, Respiratory Rate, Cytokines

## Abstract

**Introduction:**

Severe coronavirus disease 2019 (COVID-19) is characterised by hyperinflammatory state, systemic coagulopathies, and multiorgan involvement, especially acute respiratory distress syndrome (ARDS). We here describe our preliminary clinical experience with COVID-19 patients treated via an early initiation of extracorporeal blood purification combined with systemic heparinisation and respiratory support.

**Methods:**

Fifteen patients were included; several biomarkers associated with COVID-19 severity were monitored. Personalised treatment was tailored according to the levels of interleukin (IL)-6, IL-8, tumour necrosis factor alpha, C-reactive protein (CRP), neutrophil-to-lymphocyte ratio, thrombocyte counts, D-dimers, and fibrinogen. Treatment consisted of respiratory support, extracorporeal blood purification using the AN69ST (oXiris®) hemofilter, and 300 U/kg heparin to maintain activation clotting time ≥ 180 seconds.

**Results:**

Ten patients presented with severe to critical disease (dyspnoea, hypoxia, respiratory rate > 30/min, peripheral oxygen saturation < 90%, or > 50% lung involvement on X-ray imaging). The median intensive care unit length of stay was 9.3 days (interquartile range 5.3-10.1); two patients developed ARDS and died after 5 and 26 days. Clinical improvement was associated with normalisation (increase) of thrombocytes and white blood cells, stable levels of IL-6 (< 50 ng/mL), and a decrease of CRP and fibrinogen.

**Conclusion:**

Continuous monitoring of COVID-19 severity biomarkers and radiological imaging is crucial to assess disease progression, uncontrolled inflammation, and to avert irreversible multiorgan failure. The combination of systemic heparin anticoagulation regimens and extracorporeal blood purification using cytokine-adsorbing hemofilters may reduce hyperinflammation, prevent coagulopathy, and support clinical recovery.

**Table:** 

Abbreviations, acronyms & symbols				
**ACT**	**= Activation clotting time**	** **	**INR**	**= International normalised ratio**
**AKI**	**= Acute kidney injury**		**IQR**	**= Interquartile range**
**ALT**	**= Alanine aminotransferase**		**IU**	**= International units**
**ARDS**	**= Acute respiratory distress syndrome**		**LDH**	**= Lactate dehydrogenase**
**AST**	**= Aspartate aminotransferase**		**LYM**	**= Lymphocyte**
**BMI**	**= Body mass index**		**MONO**	**= Monocyte**
**BSA**	**= Body surface area**		**NEU**	**= Neutrophil**
**CI**	**= Confidence interval**		**NEU/LYM**	**= Neutrophil-to-lymphocyte**
**COVID-19**	**= Coronavirus disease 2019**		**NLR**	**= Neutrophil-to-lymphocyte ratio**
**CPAP**	**= Continuous positive airway pressure**		**NMK**	**= Republic of North Macedonia**
**CRP**	**= C-reactive protein**		**PLT**	**= Thrombocyte**
**CT**	**= Computed tomography**		**RT-PCR**	**= Reverse transcription polymerase chain reaction**
**CXCL-8**	**= Chemokine (C-X-C motif) ligand 8**		**SARS‐CoV‐2**	**= Severe Acute Respiratory Syndrome Coronavirus 2**
**ECOS**	**= Extracorporeal organ support**		**SII**	= Systemic immune-inflammation index, thrombocyte*(neutrophil-to-lymphocyte)
**EO**	**= Eosinophil**			
**FIB**	**= Fibrinogen**		**SpO2**	**= Oxygen saturation**
**HCT**	**= Hematocrit**		**STROBE**	**= STrengthening the Reporting of OBservational studies in Epidemiology**
**HGB**	**= Hemoglobin**			
**ICU**	**= Intensive care unit**		**TNF-α**	**= Tumour necrosis factor alpha**
**IL**	**= Interleukin**		**WBC**	**= White blood cell**

## INTRODUCTION

The current coronavirus disease 2019 (COVID-19) pandemic is manifesting itself as an unprecedented threat to the global population. The outbreak of the severe acute respiratory syndrome coronavirus 2 (SARS‐CoV‐2) started in Wuhan, Hubei Province, People's Republic of China^[1]^. Since then, it has spread in a rapid, deadly pace throughout the world instigating the World Health Organization to classify COVID-19 as a global epidemic on February 28, 2020^[2]^.

Severe COVID-19 is characterised by (infectious) pneumonia; complications typically include acute respiratory distress syndrome (ARDS)^[3]^. COVID-19 has also been linked with acute cardiac injury^[4,5]^, kidney malfunction^[6]^, and secondary infections^[7]^.

COVID-19 progression is associated with dysregulated immunity, commonly referred to as cytokine storm^[8]^, in particular, aberrant interleukin (IL)-6 levels^[9-11]^ that promote numerous pathological downstream effects. Hyperinflammation is a well-established trigger of multiorgan failure, *e.g*., acute kidney injury (AKI). Moreover, recent reports point to a link between hyperinflammation and COVID-19-induced coagulopathy^[12,13]^ as a result of increased production of clotting factors by the liver^[14]^.

Despite several lines of evidence pointing to a potential clinical benefit of controlling hyperinflammation triggered by COVID-19^[8]^, management of COVID-19 remains mostly supportive built around continuous respiratory support^[15-18]^.

To this end, considering the underlying immunological character of COVID-19 and the high risk of SARS-CoV-2 hyperinflammation to trigger ARDS, hypercoagulability, and AKI, we have established a treatment protocol for COVID-19. We follow selected biochemical, immunological, and coagulation risk factors to tailor therapy; our approach centres around the 1) early initiation of blood purification using the oXiris® (AN69ST) filter^[19,20]^, 2) systemic heparinisation, and 3) respiratory support, continuous positive airway pressure (CPAP), and physical therapy^[21]^.

With this initial report, we present a preliminary overview of biochemical, immunological, inflammatory, and coagulation biomarkers assessed, and offer insights into their correlations with clinical status. Finally, we report the early results in regards to treatment outcome.

## METHODS

**Fig. 1:**
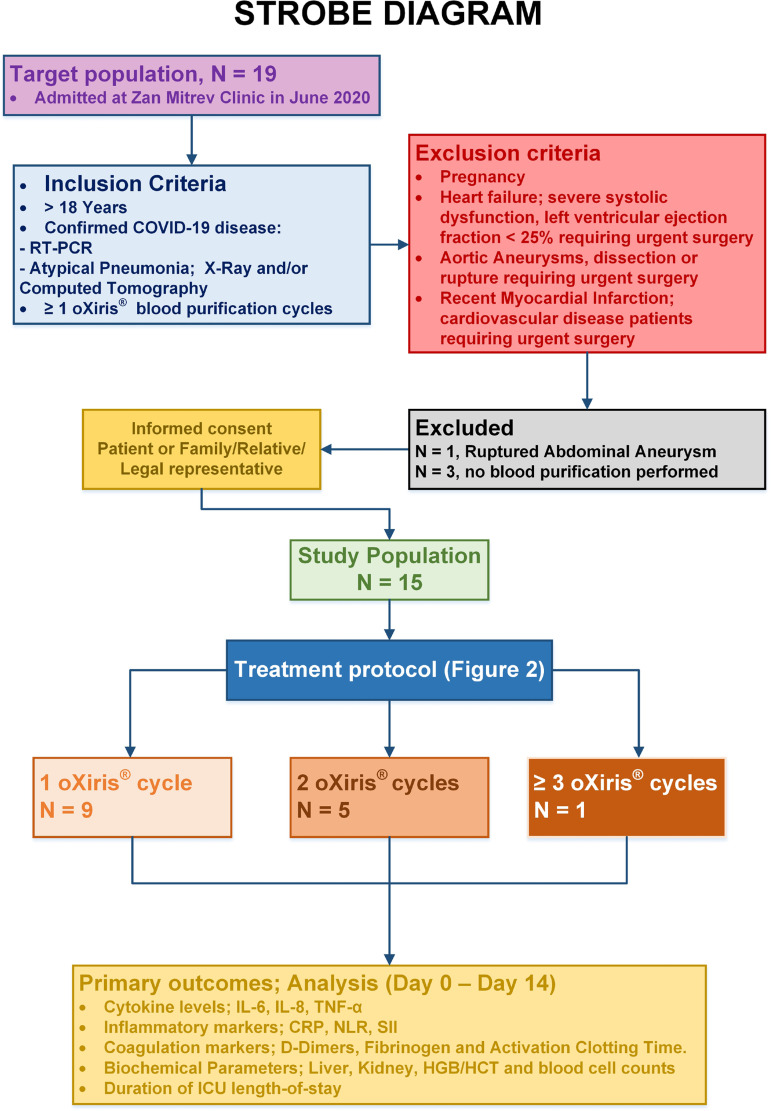
STrengthening the Reporting of OBservational studies in Epidemiology (STROBE) diagram. COVID-19=coronavirus disease 2019; CRP=C-reactive protein; HGB/HCT=hemoglobin/hematocrit; ICU=intensive care unit; IL=interleukin; NLR=neutrophil-to-lymphocyte ratio; RT-PCR=reverse transcription polymerase chain reaction; SII=systemic immune-inflammation index, thrombocyte*(neutrophil-to-lymphocyte); TNF-α=tumour necrosis factor alpha

This single-centre case series included 15 consecutive patients with confirmed COVID-19 treated in June 2020. The study designed is presented in the STrengthening the Reporting of OBservational studies in Epidemiology, or STROBE, diagram (Figure 1).

Patients were classified according to their clinical presentation in four severity degrees:

1. Mild cases
  The clinical symptoms are mild, with no apparent sign of pneumonia on imaging.
2. Moderate cases
  Showing fever and respiratory symptoms with radiological findings of pneumonia.
3. Severe cases
  A. Respiratory distress (30 breaths/min).
  B. Oxygen saturation (SpO_2_) < 90% at rest.
  C. Arterial partial pressure of oxygen (or PaO_2_)/fraction of inspired oxygen (or FiO_2_): 300 mmHg (1 mmHg = 0.133 kPa).
  *Cases with chest imaging that show lesion > 50% progression within 24 hours shall be managed as severe cases.*
4. Critical cases
  A. Respiratory failure requiring mechanical ventilation.
  B. Shock.
  C. Organ failure that requires intensive care unit (ICU) care.
    Inclusion Criteria
    - Written or temporary verbal informed consent.
    - Adults > 18 years.
    - Confirmed COVID-19 pneumonia using reverse transcription-polymerase chain reaction (RT-PCR), X-ray, and/or computed tomography.
    Exclusion Criteria:
    - Pregnancy.
    - Heart failure; severe systolic dysfunction, left ventricular ejection fraction < 25% requiring urgent surgery.
    - Aortic aneurysms, dissection, or rupture requiring urgent surgery.
    - Recent myocardial infarction; cardiovascular disease patients requiring urgent surgery

### Ethics Approval and Consent to Participate

The local ethical committee of the Zan Mitrev Clinic reviewed and approved the clinical practice, treatment procedures described, and the results reported in this manuscript and approved the submission (#EBPZ.357). Trial registration: ClinicalTrials.gov, NCT04478539. Registered 14^th^ of July 2020 - Retrospectively registered, https://clinicaltrials.gov/ct2/show/NCT04478539

### Consent for Publication

Written or temporary verbal informed consent was obtained from all patients for publication of this manuscript and any accompanying images; the use of all health and medical information for scientific research and manuscript preparation was approved. A copy of the written consent is available for review by the Editor-in-Chief of this journal.

### Availability of Data and Material

All original data described in this case report can be submitted for evaluation upon reasonable request.

### Biochemistry Analysis

Blood samples were collected from each patient at ≥ 1 x every 24 hours for routine blood analysis and to assess the treatment effects: white blood cell (WBC) count, lymphocyte count, neutrophil count, thrombocyte (PLT) count, monocyte count, and eosinophil count were determined as well as the neutrophil-to-lymphocyte (NEU/LYM) ratio (NLR) and the systemic immune-inflammation index PLT*(NEU/LYM). Moreover, blood biochemistry parameters such as Na^+^, K^+^, aspartate aminotransferase, alanine aminotransferase, bilirubin, urea, C-reactive protein (CRP), as well as procalcitonin and lactate dehydrogenase (LDH) were assessed using Siemens ADVIA Centaur XP Immunoassay System.

Data on coagulation parameters were obtained from all patients; coagulation tests included D-dimers, fibrinogen (FIB), and international normalised ratio. Tests were performed using a Sysmex CA-600 automatic coagulation analyser.

### Luminex

Analyses of human cytokines IL-6, IL-8/chemokine (C-X-C motif) ligand 8 (CXCL-8), and tumour necrosis factor alpha (TNF-α) in serum samples were performed using the Human Magnetic Luminex® assay (R&D Systems, United States of America), according to the manufacturer's instructions. The measurements were performed in triplicates using a Luminex® 100/200 System.

### Statistical Analysis

Categorical parameters were summarised as absolute numbers and percentages. Continuous data are shown as mean ± standard deviation; alternatively, non-parametric data are presented as median + interquartile range (IQR). Continuous variables were evaluated using the D'Agostino-Pearson normality test. The data were analysed with the statistical program GraphPad Prism, version 7.03.

### Treatment

**Fig. 2:**
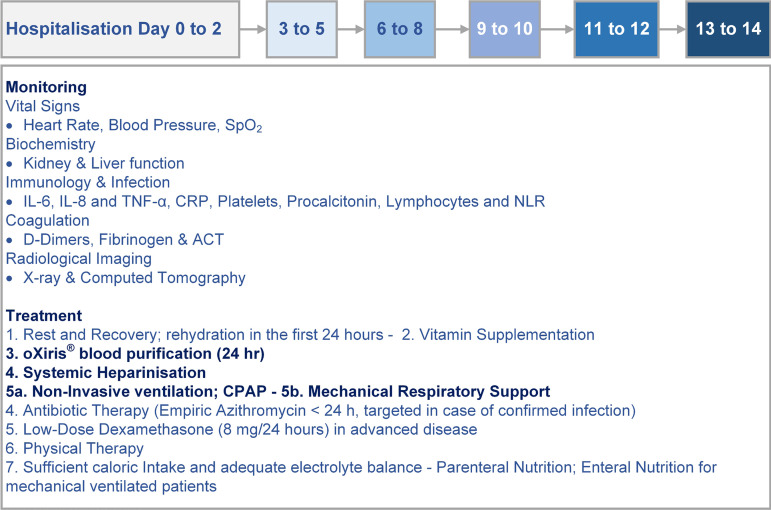
Treatment protocol for coronavirus disease 2019 patients. In addition to blood purification, systemic heparinisation, and physical therapy under continuous positive airway pressure (CPAP), the general care protocol consisted of rest and recovery; sufficient caloric intake and adequate electrolyte balance; aggressive rehydration in the first 24 hours; parenteral, enteral nutrition for mechanically ventilated patients; low-dose Dexamethasone therapy (8 mg/24 hours), antibiotic therapy, and biochemical and chest X-ray imaging for monitoring. ACT=activation clotting time; CRP=C-reactive protein; IL=interleukin; NLR=neutrophil-to-lymphocyte ratio; SpO2=oxygen saturation; TNF-α=tumour necrosis factor alpha

The treatment protocol is shown in Figure 2 and follows practice safety recommendations, treatment strategies, and up-to-date sepsis management guidelines^[22-25]^.

The multidisciplinary care and therapeutic approach consist of the early initiation of blood purification using the AN69ST (oXiris®) hemofilter, initiated within 4-12 hours of admission and high-dose heparinisation. We opt for an aggressive non-invasive respiratory therapy, including CPAP on-mask and physical therapy in an attempt to avoid mechanical ventilation. In the case of secondary infections, we administer targeted antibiotic therapy.

### Extracorporeal Organ Support (ECOS) and Blood Purification

The Prismaflex® oXiris® system was mounted in the ICU and connected 4-12 hours after admission upon establishing control of the haemostasis, activation clotting time (ACT) of 180 secs. The patient is connected to the Prismaflex® oXiris® system via a double-lumen catheter placed in the femoral vein or vena subclavia.

Flow rates were maintained as follows: effluent dose 35 mL/Kg/h, dialysate 14-16 mL/Kg/h, blood 150 mL/min, replacement fluid 16-18 mL/Kg/h; patient fluid removal is tailored to the individual's volume status ≈ 100-250 mL/h. Blood purification is initiated within 4-12 hours of admission, and the oXiris^®^ ECOS modality was chosen according to the patient's kidney function: continuous venovenous hemofiltration, continuous venovenous hemodiafiltration, or slow continuous ultrafiltration.

### Heparinisation

An initial 25000 international units (IU) bolus injection (≈ 300 IU/kg) followed by continuous infusion of 300 IU/kg dissolved in physiological buffer (0.9% sodium chloride) administered at 6-8 mL/h flow rate; target ACT ≥ 180 s during hospitalisation.

### Respiratory Support

Oxygen therapy: Patients with severe symptoms should receive nasal cannulas or oxygen masks and timely assessment of respiratory distress and/or hypoxemia should be performed.

Non-invasive ventilation: CPAP on mask for patients with SpO_2_ 86-90%; prone position.

Invasive mechanical ventilation: Lung protective ventilation strategy, namely low tidal volume (4-6 ml/kg of ideal body weight) and low level of airway platform pressure (< 30 cmH_2_O) should be used to perform mechanical ventilation to reduce ventilator-related lung injury.

While the airway platform pressure is maintained at 30 cmH_2_O, high positive end-expiratory pressure can be used to keep the airway warm and moist. Sedation and muscle relaxants were used according to the clinical condition and preferably in a prone position. Furthermore, anaesthesia regimens are tailored to promote early weaning from mechanical ventilation.

## Antibiotic Therap

Empiric administration of Azithromycin in the first 48 hours; antibiotic therapy is discontinued, switched to targeted according to the antimicrobial susceptibility testing^[26,27]^.

## Medical Therapy

Individual medical therapy was continued according to the patient's pre-existing conditions and comorbidities.

## RESULTS

A total of 15 patients with confirmed SARS-CoV-2 infection manifesting as COVID-19 were treated at our clinic in June 2020. Table 1 shows the basic patient characteristics. Of the 15 cases, two were females - the mean age of the cohort was 60.2 years (range 27-83). The patients were referred to us from peripheral hospitals across the country.

**Table 1:** Basic patient characteristics.

Age (years)	60.2±12.8
Female gender (%)	2 (13%)
BSA (m^2^)	1.9±0.14
BMI	26.7±2.4
Diabetes	2
Hypertension	6
Obesity (BMI > 35 Kg/m^2^)	2
Glucose (mmol/L)	6.6 (5.7-12.8)
Creatinine (µmol/L)	70.9±14.3
Urea (mmol/L)	4.5 (3.1-6.2)
Aspartate transaminase (U/L)	58.9±23.2
Alanine transaminase (U/L)	75.1±40.4
Bilirubin (µmol/L)	6.68±0.73
Lactate dehydrogenase (U/L)	330.5 (258.8-453.5)
Hemoglobulin (g/dL)	13.2 (12.3-14.0)
Hematocrit (%)	37.90 (36.30-40.40)
Na^+ ^(mmol/L)	137.3±2.6
K^+ ^(mmol/L)	3.8±0.54
Procalcitonin (ng/mL)	0.07±0.04
C-reactive protein (mg/mL)	74.1 (55.1-127.8)
White blood cell counts (*10^3^ counts/µL)	6.3 (2.9-10.1)
Platelets (*10^3^ counts/µL)	140 (108 to 208)
NEU (%)	83.11 (64.3-89.2)
LYM (%)	9.8 (7.3-21.5)
MONO (%)	3.3 (2.6-6.9)
EO (%)	0.1 (0.04-0.44)
NLR (*10^3^ counts/µL)	8.3 (3.5-12.2)
Systemic immune-inflammation index	1311 (406.2-2791)
D-dimers (ng/mL)	790.0 (395.0-1980)
Fibrinogen (g/L)	7 (3.6-8)

BMI=body mass index; BSA=body surface area; EO=eosinophil;MONO=monocyte; NLR=neutrophil (NEU)-to-lymphocyte (LYM) ratio

**Fig. 3:**
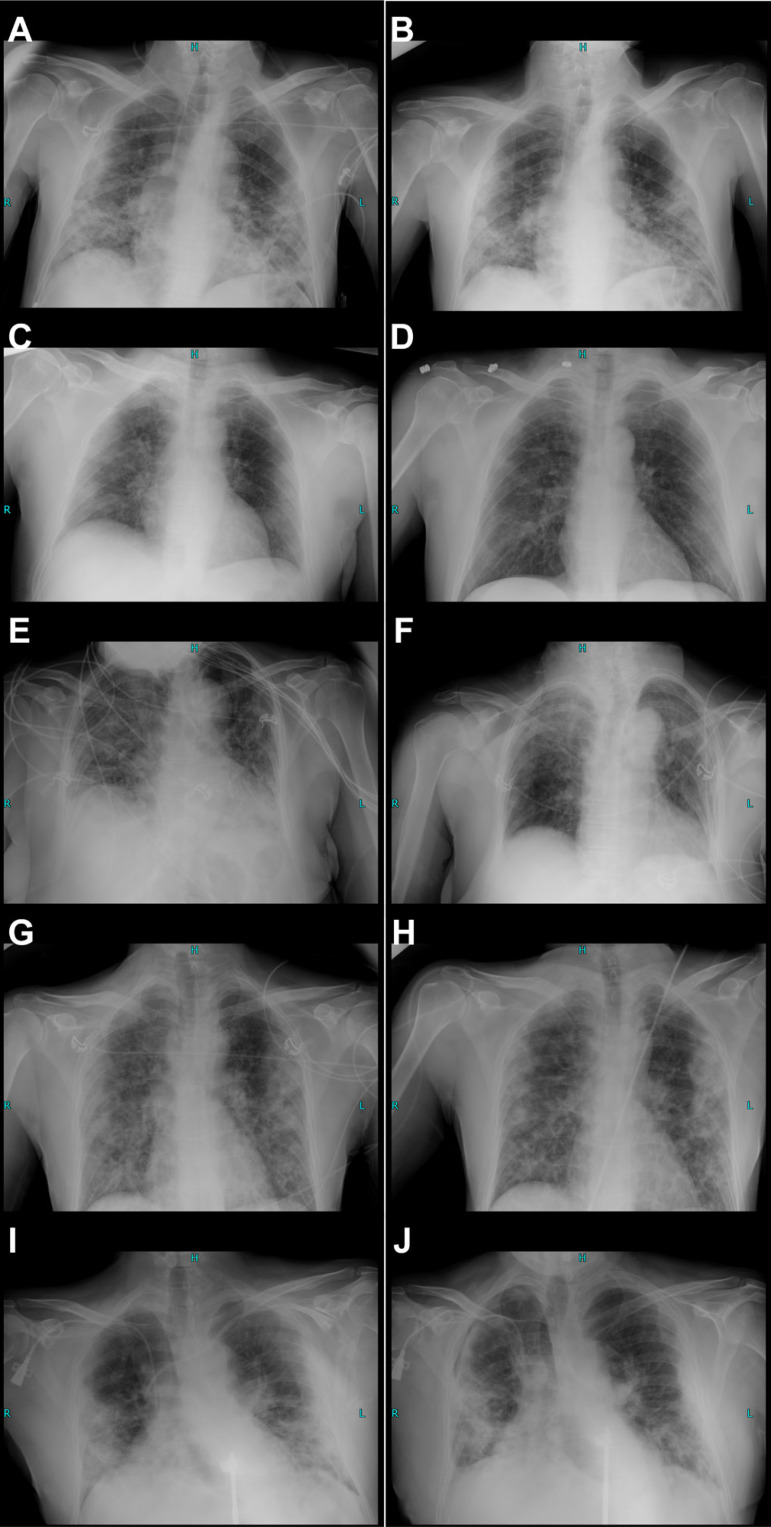
Pre- and post-treatment X-ray images. (A) 62-year-old male (Figure 6M) was admitted with oxygen saturation (SpO2) of 93%, fatigue, and breathing difficulties. Before his admission, he had several episodes of high body temperature (39 °C). Chest radiography on admission showed bilateral patchy reticular areas of opacifications, perihilar and peripheral distribution with lower zone predominance, and subsegmental atelectasis in the mid-zone of the left lung. (B) Control X-ray showing minor regression of baseline findings. (C) A 67-year-old male with severe coronavirus disease 2019 (COVID-19) was admitted with breathing difficulties, SpO2 of 85%, and Staphylococcus aureus (cytokine profile shown in Figure 6F); we noted patchy bilateral areas of opacifications with lower zone predominance, right perihilar, and left peripheral distribution. (D) We discharged him after 10 days with significantly improved SpO2 96% and regression of X-ray findings. (E) We admitted a 70-year-old hypertensive febrile (38 ºC) female (Figure 6I) with SpO2 of 85%, dyspnoea, tachypnoea, and COVID-19 pneumonia; we observed bilateral reticulonodular areas of opacifications with perihilar and peripheral distribution, with consolidation in the upper right lung and evidence of right pleural effusion. Her condition was complicated because of Klebsiella pneumoniae, Streptococcus beta haemolyticus co-infection in respiratory samples, and vancomycin-resistant Enterococcus in urine samples taken within 24 after admission. At discharge, we observed minimal regressions in findings of consolidation and resolution of the right pleural effusion (F). Panel (G) shows the first X-ray image taken of a 51-year-old male (Figure 6J); chest X-ray findings point to bilateral perihilar and peripheral extensive patchy opacifications and a prominent zone of consolidation in the mid- and upper peripheral section, the left lobe was more affected. (H) Treatment resulted in the normalisation of peripheral oxygen saturation values. Still, X-ray images suggested a minor progression of initial findings; non-resolving bilateral consolidations with bigger consolidation zone in the left upper peripheral lung. Panel (I) shows patchy bilateral consolidations, perihilar and peripheral distribution, of a 50-year-old male (Figure 6B) admitted with a SpO2 of 92% and previous episodes of high body temperature. He received two cycles of oXiris® blood purification and on the explicit, consented request of his family and relatives he was also treated with 8 mg/kg Tocilizumab given over 120 minutes via intravenous infusion. (J) Chest X-rays showed progression; bilateral consolidations, pleural effusion, and small apical pneumothorax in the right lobe. He was initially treated with Azithromycin which was adapted to Ciprofloxacin after multiplex reverse transcription-polymerase chain reaction identified Staphylococcus aureus and Klebsiella pneumoniae; he was discharged after 15 days.

Primary symptoms reported were dyspnea, fever, and low peripheral saturation; 10 cases presented with severe disease; all patients had advanced COVID-19 pneumonia (Figure 3).

Patients presented with elevated levels of CRP (74.1 mg/L; IQR 55.10-127.8), mild thrombocytopenia (140*10^3^ counts/µL; IQR 108-208), and increased values of D-dimers (790.0 ng/mL; IQR 395-1980) and FIB (5.8±2.4 g/L). LDH and NLR were high at admission with values of 330.5 IU (IQR 258.8-453.5) and 8.3 (IQR3.5-12.2), respectively (Table 1). Two patients were intubated within 24 hours of admission; both patients did not recover and died on the 5^th^ and 26^th^ hospitalisation day, respectively; both cases developed severe ARDS and multiorgan failure.

The other patients were discharged after, on average, 9.3 days (IQR 5.3-10.1) of intensive care in our COVID-19 center.

**Fig. 4:**
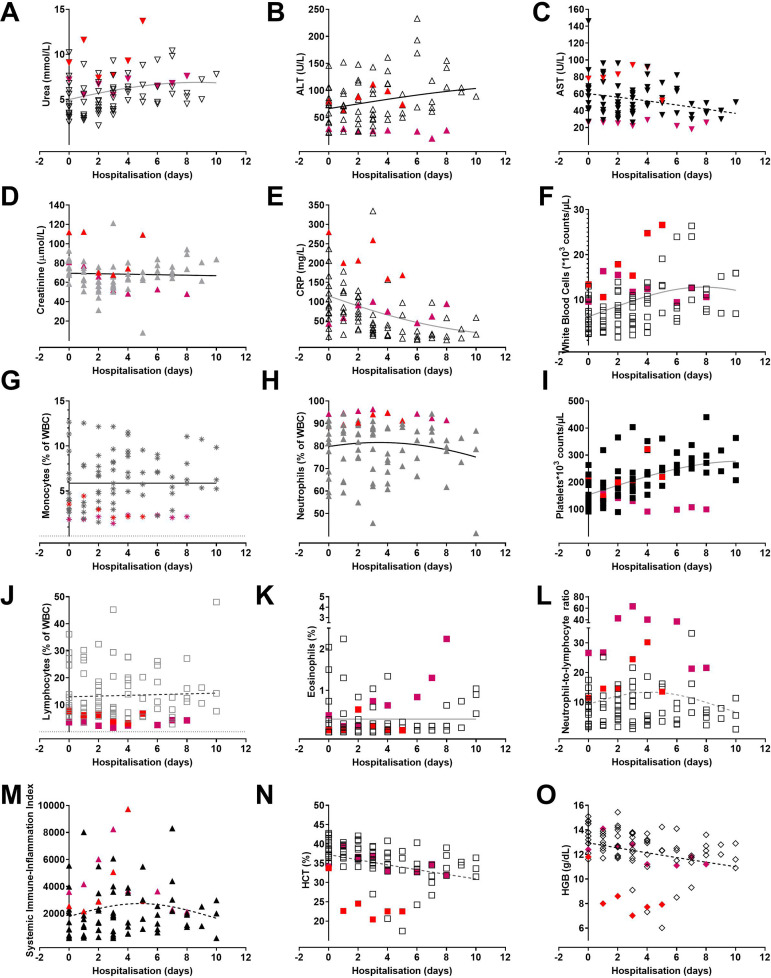
Biochemical parameters during hospitalisation. Graphs present an overview of selected biochemical parameters monitored for coronavirus disease 2019 severity. Red lines show a general trendline during hospitalisation. The red coloured symbols pertain a patient who succumbed as a result of acute respiratory distress syndrome. The coloured (red #1) (purple #2) symbols show the values for the two mortality cases. ALT=alanine aminotransferase; AST=aspartate aminotransferase; CRP=C-reactive protein; HCT=hematocrit; HGB=hemoglobin; WBC=white blood cell

**Fig. 5:**
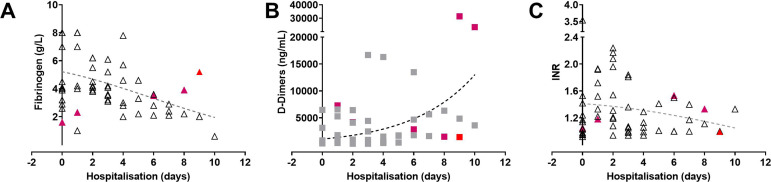
Analysis of coagulation markers. Patients receive an initial 25000 international units (IU) bolus injection (≈ 300 IU/kg) followed by continuous infusion of 300 IU/kg dissolved in physiological buffer (0.9% sodium chloride) administered at 6-8 mL/h flow rate; target activation clotting time ≥ 200 s during hospitalisation. Patients' coagulation statuses were tracked by evaluating fibrinogen, D-Dimers, and the international normalised ratio (INR). The coloured (red #1) (purple #2) symbols show the values for the two mortality cases.

Treatment led to a gradual normalisation of biochemical parameters (Figure 4); in particular, we observed a linear trend (r=0.40, 95% confidence interval [CI] 0.21 to 0.57; *P*<0.0001) between platelet numbers, WBC (r=0.37, 95% CI 0.18 to 0.54; *P*=0.0003), and the clinical picture during hospitalisation suggesting that an increase of PLT was associated with recovery. A similar trend was observed for the WBC. In contrast, clinical recovery was associated with a decrease in FIB levels (r=-0.45, 95% CI -0.63 to -0.21; *P*=0.0004) and CRP (r=0.39 95% CI -0.57 to -0.20) (Figure 5A).

**Fig. 6:**
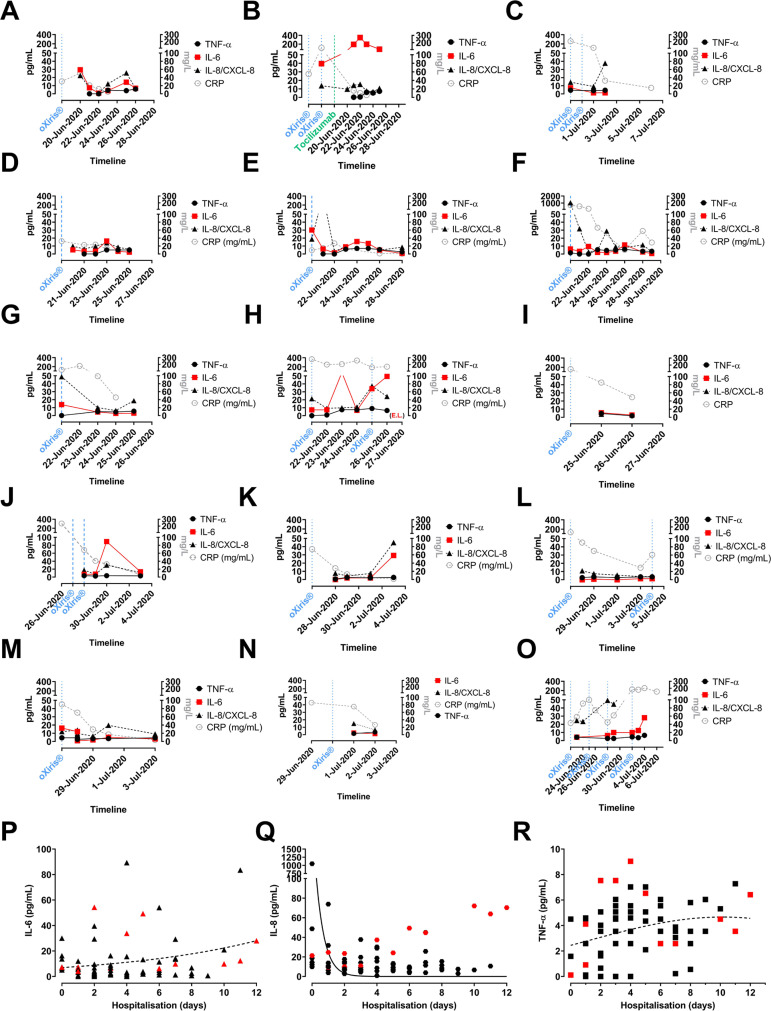
Inflammatory mediator analysis; systemic levels of interleukin (IL)-6, IL/chemokine (C-X-C motif ) ligand 8 (CXCL-8), and tumour necrosis factor alpha (TNF-α). Individual cytokine profile (A - O) IL-6, IL-8, and TNF-α are plotted on the left y-axis (pg/mL), and C-reactive protein (CRP) is plotted on the right y-axis (mg/L). The start of oXiris® hemofiltration 24-cycle is shown on the x-axis. One patient (Panel B) also received Tocilizumab (= anti-IL-6 receptor mAb). Cytokine data are plotted on the left y-axis; CRP (grey checkered line) values are plotted on the right y-axis. Panels (P, Q, and R) show combined data during hospitalisation for IL-6, IL-8, and TNF-α. The coloured (red #1) (purple #2) symbols show the values for the two mortality cases.

IL-6 is the primary cytokine leading to hepatic CRP production; we observed that early initiation of oXiris^®^ blood purification was associated with stable or decreasing levels of IL-6, IL-8, and TNF-α which in turn led to a gradual reduction of systemic CRP levels across the whole cohort (Figures 4 and 6).

The treatment approach led to an improvement in SpO_2_, a decrease of inflammatory mediators, and an increase in the number of PLT.

In one particular case, a 50-year-old male admitted with a SpO_2_ of 92% on 2L of oxygen (Figure 2I and J) with previous episodes of high body temperature received, in addition to the two cycles of oXiris^®^ blood purification, 8 mg/kg of Tocilizumab given over 120 minutes via intravenous infusion (Figure 6B). The latter was administered on the explicit, consented request of his family. Administration of IL-6r blocking antibody led to a transient spike of IL-6 levels as reported before^[28]^. He was also treated with Azithromycin, which was adapted to Ciprofloxacin after multiplex RT-PCR identified Methylin-resistant *Staphylococcus aureus* and *Klebsiella pneumoniae*; he was discharged after 15 days.

In some cases, the clinical course was complicated because of bacterial co-infections; a case of a 56-year-old male with dyspnea, SpO_2_ of 90% on room air, and a body temperature of 38 °C at admission was challenging due to a *Klebsiella pneumoniae* infection.

The same pathogen was detected in a 70-year-old female. We also confirmed *Streptococcus beta haemolyticus* in her respiratory samples and vancomycin-resistant *Enterococcus* in urine samples taken within 24 hours after admission. We successfully treated her with two cycles of blood purification and antibiotics consisting of Azithromycin and Ampicillin/Sulbactam.

Another male presenting with high fever (38.8 °C), dry cough, dyspnea, and SpO_2_ of 85% had a co-infection of *Streptococcus pneumoniae* in his throat swabs detected using RT-PCR. We treated him with two cycles of oXiris^®^ blood purification and Azithromycin. He was discharged after eight days with markedly recovered symptoms: CRP level was 6.4 mg/L, WBC count of 4.5x10^3/µL, and normalised platelet count was 186*10^3^/µL.

The first mortality case involved an 83-year-old male with dyspnoea, tachypnoea, and extremely low SpO_2_ (65%) despite 6L oxygen, suggesting ARDS. Biochemical analysis revealed significant abnormalities; CRP was 279.9 mg/L, and LDH was 671 U/L. He was immediately placed on extracorporeal blood purification; we also discovered *Streptococcus beta haemolyticus* in his throat, and nasal swab detected it. Targeted therapy with Ampicillin/Sulbactam was initiated. However, despite intensive treatment including mechanical ventilation and a total of three cycles of oXiris^®^ hemofiltration, the patient's condition deteriorated over the next days as he developed multiorgan failure; he passed away five days after his admission (Figure 6H).

The 2^nd^ mortality case pertained to a 73-year-old male admitted with dyspnoea, tachypnoea, severely reduced SpO_2_ (< 70%) on room air and elevated LDH at 527 U/L. Despite two cycles of oXiris^®^ blood purification, SpO_2_ levels were not improving. We were able to stabilise his condition with mechanical respiratory support. His condition was sensitive due to the discovery of *Klebsiella pneumoniae* in his bronchial secretion. For this reason, we switched antibiotherapy to include Ampicillin/Sulbactam. Still, despite systemic heparinisation, the levels of D-dimers (Figure 5B, purple coloured symbols) were increased to 31400 ng/mL on the 9^th^ hospitalisation day. Towards the end of his 4^th^ oXiris® cycle, we observed notable improvements, and we were able to extubate him in the next 48 hours (Figure 6O). However, within 48 hours his condition suddenly worsened necessitating re-intubation. Additional cycles of blood purification were unsuccessful in rescuing his clinical situation, and he succumbed to complications related to ARDS on the 26^th^ day on the ICU.

In summary, a treatment approach based on early initiation of blood purification using the AN69ST (oXiris^®^) hemofilter, systemic heparinisation, and respiratory support may support clinical recovery in moderate to severe cases of COVID-19.

## DISCUSSION

We present with this work our initial case series of 15 COVID-19 patients treated with early initiation of extracorporeal blood purification using the oXiris® (AN69ST) hemofilter, systemic heparinisation, and respiratory support; we monitored several biochemical, immunological, inflammatory, and coagulation biomarkers to tailor therapy to the individual requirements.

The first cases of COVID-19 in the Republic of North Macedonia (NMK) were confirmed in early March 2020. The country has seen a sudden rise in the confirmed cases since restrictions were lifted in May 2020; the number of cases is slowly outnumbering the national ICU-bed capacity.

As of July 28^th^, the current COVID-19 pandemic has resulted in 466 deaths over a population of roughly 2.0 million. These numbers echo the global statistics, with 16.5 million confirmed cases of COVID-19 worldwide and an estimated mortality rate of 3.7%^[3]^. About 5% of the infected population will develop advanced disease requiring intensive care, often necessitating ECOS therapies. Of this critically ill subgroup, the mortality rate is high 40-50%^[6]^.

**Supplemental Figure 1:**
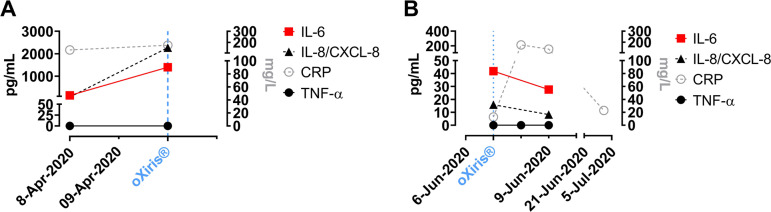
(A) A 73-year-old male coronavirus disease 2019 (COVID-19) patient was admitted for urgent surgery; at admission, he was in hemorrhagic shock. Computed tomography (CT) revealed a ruptured infrarenal 10-cm aneurysm of the abdominal aorta. Radiography confirmed bilateral pneumonia. Despite successful surgery, his clinical condition was unstable, requiring increasing catecholamine support. Extracorporeal blood purification was initiated on the 3rd postoperative course. The approach was unable to reverse the clinical deterioration, he developed multiorgan failure and passed away in the early hours of the 4th postoperative day. (B) A 55-year-old patient with confirmed reverse transcription polymerase chain reaction for Severe Acute Respiratory Syndrome Coronavirus 2 (SARS-CoV-2) and mild COVID-19 symptoms was hospitalised at our emergency room with abdominal complaints, most pronounced in the epigastric region, CT pointed to a ruptured infrarenal aneurysm of the abdominal aorta with a diameter of about 7 cm. The aneurysm was treated with an AlboGraft® prosthesis followed with a blood purification cycle on the 1st postoperative day. In the subsequent five days, he tested twice negative for SARS-CoV-2. He was discharged on the 6th postoperative day in stable condition; follow-up after 30 days of the procedure confirmed his clinical recovery. CRP=C-reactive protein; CXCL-8=chemokine (C-X-C motif ) ligand 8; IL=interleukin; TNF-α=tumour necrosis factor alpha

We report here our initial case series; it is a relatively small cohort compared to global numbers. The reason being that our clinic was initially designated a "clean" hospital and allowed only to perform cardiovascular emergency procedures. Confirmed COVID-19 patients between March and June were referred to the public clinic of infectious diseases. During the first months of the outbreak in NMK, we operated on two COVID-19 patients with ruptured abdominal aorta aneurysms; the first case presented an extremely critical condition and succumbed to his condition on the 2^nd^ postoperative day. The second case was successfully treated with a protocol resembling approach described in this report (Supplementary Figure 1). He was discharged after six days. Follow-up at 30 days pointed to a gradual normalisation of several inflammatory biomarkers; for instance, CRP was reduced to 29.9 mg/L from the initial 175.6 mg/L at admission.

The role of lung injury in COVID-19 is well-established; however, recent observations point to a high risk for AKI in COVID-19 patients^[6]^, but also hypercoagulability^[12]^. Several lines of evidence have implicated a role for pro-inflammatory cytokines in the pathology of COVID-19, especially in severe cases. Ruan et al.^[7]^ described that the critically-ill patients had higher systemic levels of IL-2, IL-7, IL-10, granulocyte colony-stimulating factor, interferon-gamma-inducible protein-10, monocyte chemotactic protein 1, macrophage inflammatory protein-1A, TNF-α, and IL-6. Aberrant IL-6 levels were indicative of an adverse outcome. Another marker associated with disease severity and adverse outcomes is the NLR^[29,30]^. In addition, hypercoagulability is now considered as one of the hallmarks of COVID-19 progression with both D-dimers^[31]^ and FIB levels^[32]^ suggested having predictive power in establishing disease severity^[13]^.

The intensive monitoring of the aforementioned parameters (Figures 4, 5, and 6) guides our clinical practice and allows us to tailor our treatment to the acute needs of the patient. Treatment focuses on limiting lung injury and on promoting physiological breathing using daily intermittent physical therapy regimens combined with CPAP-ventilation and prone position. Secondly, hypercoagulability and possibility of thromboembolism were countered through systemic administration of high dosages of heparin to maintain ACT > 180 seconds. It is noteworthy to mention that even bolus dosages of 25000 IU were not sufficient to reach ACT values of > 200 seconds, pointing to severe dysregulation of the coagulation cascade in COVID-19 patients. Thirdly, hyperinflammation was controlled using oXiris^®^ hemofilter based extracorporeal blood purification.

Control of systemic levels of cytokines (IL-6, IL-8/CXCL-8/TNF-α) (Figure 6) was achieved using the Prismaflex^®^ system (Baxter International Inc. Deerfield, Illinois) mounted with the oXiris^®^ hemofilter. The oXiris^®^ filter is a hollow fibre acrylonitrile and methanesulfonate (AN69ST) membrane^[19]^ that removes larger molecular weight molecules. Approved first in Europe in 2009, its initial CE-marked indication was extended in 2017 for patients who require blood purification, including those requiring continuous renal replacement therapy, and in conditions with excessive endotoxin and inflammatory mediator levels. The system also received emergency Food and Drug Administration authorisation for COVID-19 treatment in April^[33]^.

The oXiris® filter uses a modified AN69ST membrane and has an affinity for both endotoxins and cytokines. The modified oXiris® membrane has three-fold more polyethyleneimine for optimal endotoxin adsorption. Additional (10-fold) higher amount of immobilised heparin efficiently reduces thrombogenicity^[34]^. It has shown a superb capacity to adsorb cytokines and endotoxins^[35]^ control abnormal levels of systemic cytokines^[36]^ and improve haemodynamic parameters^[37,38]^. To this end, our COVID-19 treatment bundle is based on the use of oXiris® blood purification to counter the multidimensional inflammatory attack on the body triggered by the SARS-CoV-2.

Our treatment approach is built on our previous experience^[39]^ and our ongoing partnerships with European experts on the treatment of sepsis and related infections diseases^[40]^.

Blood purification has been evaluated in mechanically ventilated COVID-19 cases^[41]^ and patients on extracorporeal membrane oxygenation^[42]^. Both groups reported promising results, an effective reduction in pro-inflammatory cytokines and recovery in most of the treated patients.

Our results further provide empiric evidence for the effectiveness of blood purification to prevent, control and reduce hyperinflammation in COVID-19. However, our treatment approach differs on three key points: 1) the blood purification device, oXiris® *vs.* CytoSorb® ; the latter is a CE-marked device containing polymer beads to adsorb cytokines used in blood pump circuits *vs*. the modified AN69ST membrane; 2) we performed blood purification in moderate to severe cases within 4-12 hours of admission, intending to prevent disease progression and the need of mechanical ventilation; and 3) we use repetitive cycles whenever the inflammation markers were increasing.

Collectively, the work here presents a promising outlook on the treatment possibilities using a standardised procedure based on 1) control of excessive pro-inflammatory cytokines through early initiation of blood purification (Figure 6), 2) prevention of hypercoagulability through systemic heparinisation (Figure 5), and intensive physical therapy combined with CPAP-respiratory support (Figure 2).

In summary, we observed that clinical recovery was associated with an increase in PLT and WBC, whereas a decrease in CRP and FIB was observed in patients with improving clinical conditions.

Blood purification to control excessive inflammation has gained acceptance as a treatment modality for COVID-19^[20,43]^ and was successfully used in one case^[44]^.

### Limitations

Our findings propose a base for further evaluation but should be appraised with caution due to the limitations of single-centre observational studies^[45]^ and the small cohort. We provide several lines of evidence that a fully integrated digital monitoring system to guide timing, and intensity of blood purification may support clinical recovery; however, clinical trials are required to assess our findings and determine the clinical effectiveness and safety of blood purification in critically ill COVID-19 patients.

Finally, a long-term assessment is warranted to determine complete recovery, especially the resolution of COVID-19 pneumonia and recuperation of normal lung function.

## CONCLUSION

An early initiation of blood purification using the oXiris^®^ hemofilter was effective in preventing aberrant pro-inflammatory levels of COVID-19 patients. Furthermore, we observed no cases of thromboembolism which might be linked to the systemic heparinisation regimen.

Collectively, we show that real-time digital monitoring of vital signs, biochemical, immunological, and coagulation markers, and X-ray imaging in COVID-19 patients offer the opportunity to track disease severity and tailor therapy based on cytokine-hemofiltration, heparin anticoagulation, and respiratory support.

Finally, a multi-centre randomised study is warranted to adequately scrutinise the clinical effectiveness of extracorporeal blood purification in the treatment of COVID-19.

**Table:** 

Authors' roles & responsibilities	
PU	Responsible for diagnostics and patient care; drafting the work; final approval of the version to be published
DP	Responsible for diagnostics and patient care; drafting the work; final approval of the version to be published
TG	Responsible for diagnostics and patient care; final approval of the version to be published
DN	Responsible for diagnostics and patient care; drafting the work; final approval of the version to be published
DV	Responsible for diagnostics and patient care; final approval of the version to be published
MG	Responsible for diagnostics and patient care; final approval of the version to be published
ES	Responsible for diagnostics and patient care; final approval of the version to be published
SM	Responsible for diagnostics and patient care; final approval of the version to be published
LV-K	Performed the radiological examinations; final approval of the version to be published
KI	Final approval of the version to be published
SM	Responsible for cytokine analysis; final approval of the version to be published
EA	Responsible for the medical policies; and critical review of the work; final approval of the version to be published
RAR	Responsible for cytokine analysis; academic assistance; coordination of acquisition of data; analysis of data; drafting the work; final approval of the version to be published
ZM	Study director; Responsible for diagnostics and patient care; final approval of the version to be published
